# Supplementary figures and images for: Perception and demand for healthy snacks/beverages among US consumers vary by product, health benefit, and color
Source: PLoS One. 2023 Jun 16;18(6):e0287232. doi: 10.1371/journal.pone.0287232 (PMC10275438; doi:10.1371/journal.pone.0287232)

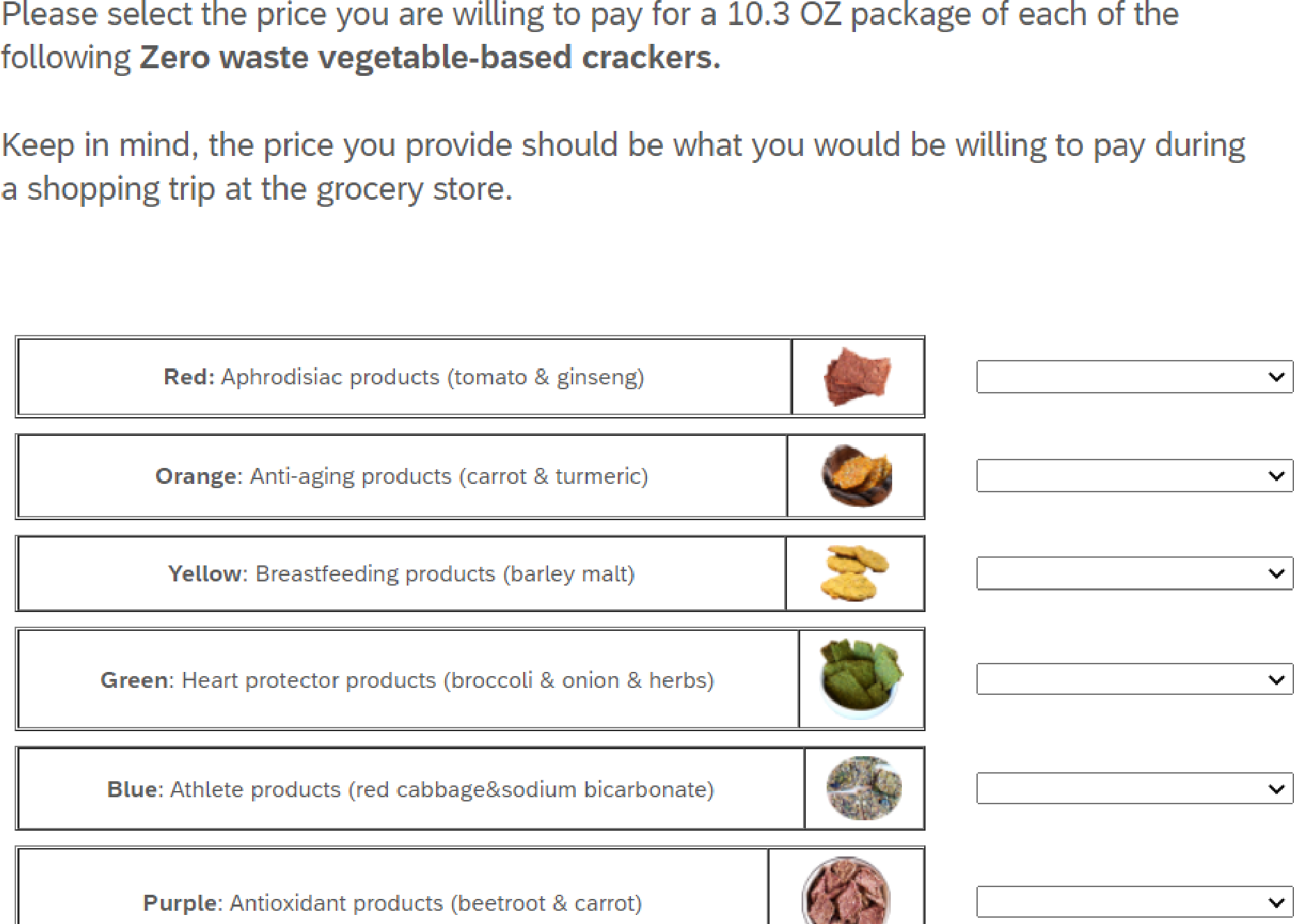

Supplement: S1 Appendix — (TIF) [file pone.0287232.s001.tif]

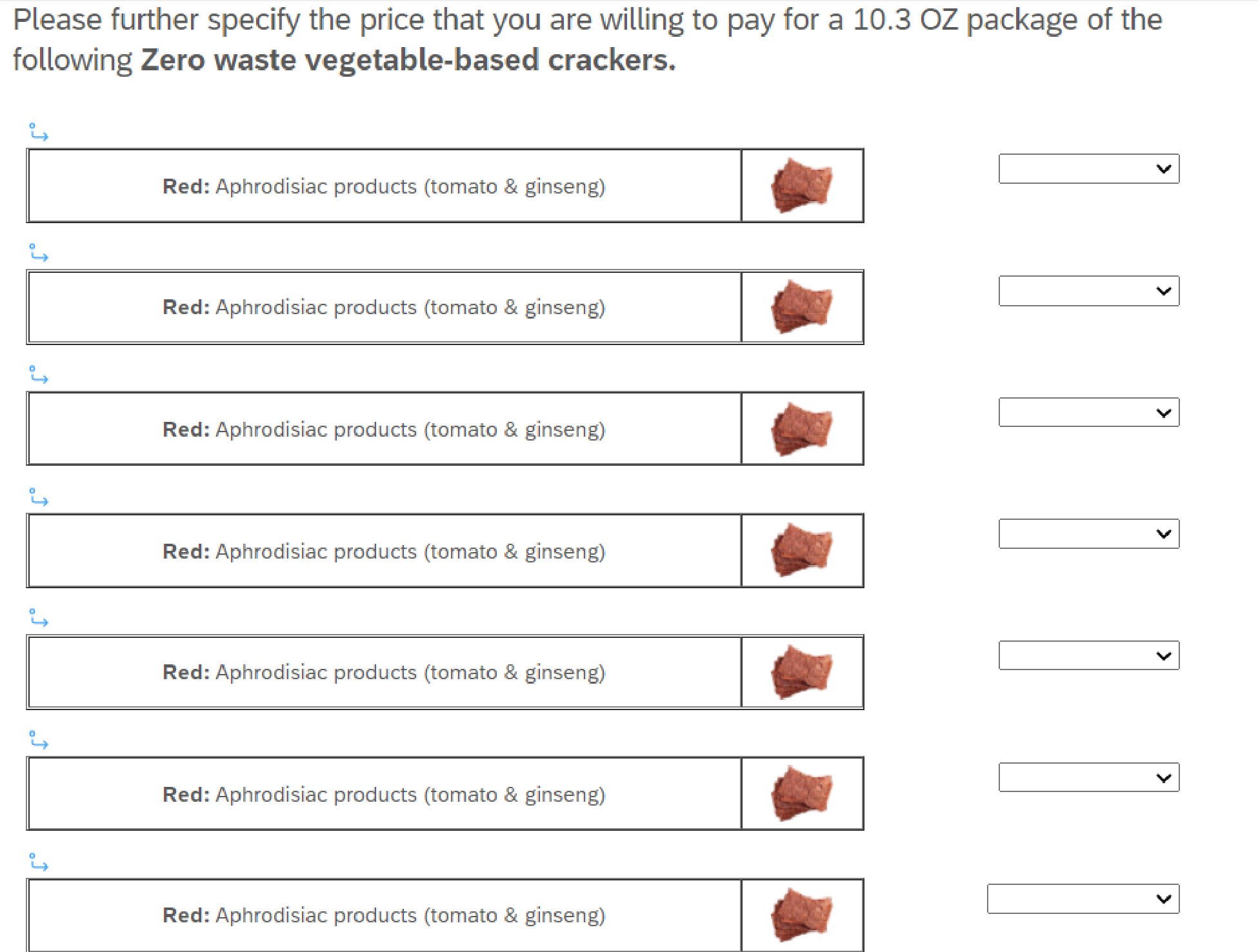

Supplement: S2 Appendix — (TIF) [file pone.0287232.s002.tif]
