# Supplementary material for: Perception and demand for healthy snacks/beverages among US consumers vary by product, health benefit, and color
Source: PLoS One. 2023 Jun 16;18(6):e0287232. doi: 10.1371/journal.pone.0287232 (PMC10275438; doi:10.1371/journal.pone.0287232)
Supplement: Attachment — Submitted filename: Response to Academic Editor and Reviewers.docx [file pone.0287232.s003.docx]

**Response to Academic Editor and Reviewers**

**Response to Academic Editor**

**Comment 1:** Please ensure that your manuscript meets PLOS ONE's style requirements, including those for file naming. The PLOS ONE style templates can be found at

[https://journals.plos.org/plosone/s/file?id=wjVg/PLOSOne_formatting_sample_main_body.pdf](https://nam10.safelinks.protection.outlook.com/?url=https%3A%2F%2Fjournals.plos.org%2Fplosone%2Fs%2Ffile%3Fid%3DwjVg%2FPLOSOne_formatting_sample_main_body.pdf&data=05%7C01%7Cg.okpiaifo%40ufl.edu%7Cae3943de1cef4355e12508db1995dfa0%7C0d4da0f84a314d76ace60a62331e1b84%7C0%7C0%7C638131903994218851%7CUnknown%7CTWFpbGZsb3d8eyJWIjoiMC4wLjAwMDAiLCJQIjoiV2luMzIiLCJBTiI6Ik1haWwiLCJXVCI6Mn0%3D%7C3000%7C%7C%7C&sdata=RoOm07XBAWemnXFTzitZVpUtB9ycZx%2Bn3A%2B760jW17I%3D&reserved=0) and

[https://journals.plos.org/plosone/s/file?id=ba62/PLOSOne_formatting_sample_title_authors_affiliations.pdf](https://nam10.safelinks.protection.outlook.com/?url=https%3A%2F%2Fjournals.plos.org%2Fplosone%2Fs%2Ffile%3Fid%3Dba62%2FPLOSOne_formatting_sample_title_authors_affiliations.pdf&data=05%7C01%7Cg.okpiaifo%40ufl.edu%7Cae3943de1cef4355e12508db1995dfa0%7C0d4da0f84a314d76ace60a62331e1b84%7C0%7C0%7C638131903994218851%7CUnknown%7CTWFpbGZsb3d8eyJWIjoiMC4wLjAwMDAiLCJQIjoiV2luMzIiLCJBTiI6Ik1haWwiLCJXVCI6Mn0%3D%7C3000%7C%7C%7C&sdata=Q8KGVoxuIKgdtWyyJfDIl98jiLUNkc5O6IqbkWveeNc%3D&reserved=0)

**Response to comment 1:** Thank you for your comment. We have formatted the title page and the manuscript headings to meet PLOS ONE’s style requirements.

**Comment 2:** Please provide additional details regarding participant consent. In the ethics statement in the Methods and online submission information, please ensure that you have specified what type you obtained (for instance, written or verbal, and if verbal, how it was documented and witnessed). If your study included minors, state whether you obtained consent from parents or guardians.

**Response to comment 2:** Thank you for your comment. We have specified the consent type obtained.

Line 104-109

“The study was approved by the Institutional Review Board at the University of Florida (IRB 201901626), and participants were provided written informed consent before answering the survey questions. The last part of the informed consent read as, “By choosing "continue" on the question below, you are indicating that you voluntarily agree to participate in this survey.” And respondents who did not select “continue” would exit the survey.”

**Comment 3:** Please upload a new copy of Figures 1, 2, 3, 4 and 5 as the detail is not clear. Please follow the link for more information: [https://blogs.plos.org/plos/2019/06/looking-good-tips-for-creating-your-plos-figures-graphics/](https://nam10.safelinks.protection.outlook.com/?url=https%3A%2F%2Fblogs.plos.org%2Fplos%2F2019%2F06%2Flooking-good-tips-for-creating-your-plos-figures-graphics%2F&data=05%7C01%7Cg.okpiaifo%40ufl.edu%7Cae3943de1cef4355e12508db1995dfa0%7C0d4da0f84a314d76ace60a62331e1b84%7C0%7C0%7C638131903994218851%7CUnknown%7CTWFpbGZsb3d8eyJWIjoiMC4wLjAwMDAiLCJQIjoiV2luMzIiLCJBTiI6Ik1haWwiLCJXVCI6Mn0%3D%7C3000%7C%7C%7C&sdata=jDZmtyYSBoEGLbxogSMi1z5DC3UrYNgIRAWBv1L5fmI%3D&reserved=0)

**Response to comment 3:** Thank you for your comment. We have made new copies of the figures using the PLOS ONE tool.

**Additional Editor comments**

**Comment 4:** Please authors, kindly attend to the comments raised by the reviewers.

**Response to comment 4:** Thank you. We have addressed all the reviewers’ comments and included details below on revisions made to address each comment raised by each reviewer.

**Comment 5:** Kindly pay attention to the justification of this current study, this needs further details. Why is this study relevant?

**Response to comment 5:** Thank you for your comment. Given the prevalence of unhealthy snacks and their associated negative health outcomes, our study contributes to efforts to identify healthy snacking options that will be appealing to consumers and economically viable. We explained this justification in the manuscript by giving a background on the problem – unhealthy snacking – and why studies like ours are needed.

To make sure that this relevance is clearer and to address your comments, we added a statement addressing our contribution to literature and efforts to improve nutrition quality.

Lines 74 – 76

“Our study contributes to this growing literature and efforts to improve nutrition quality by examining consumers’ preferences for healthy snacking options and the possible predictors of these preferences.”

Lines 84 - 91 also provide more information on our contribution to the literature and food industry/policy makers –

“To the best of our knowledge, this is the first study to measure consumer WTP for multiple novel healthy snacks and beverage products that are differentiated by appearance and health benefits. The three focus product categories chosen for this study – crackers, spreads and beverages – were selected because these are popular snacking options at social gatherings. This study provides valuable insights for food industries to develop more healthy snacks and beverages that appeal to consumers and inform marketing campaigns to promote healthy snacking. The results of this study can also benefit policymakers by providing information to guide dietary guidelines of healthy snacking recommendations.”

**Comment 6:** Also, the methodology of the work, please provide additional details.

**Response to comment 6:** Thank you for your comment. We have provided additional details about the consent type, the survey progression, and also included a table defining the statements used to capture the personality traits.

Lines 104-109

“The study was approved by the Institutional Review Board at the University of Florida (IRB 201901626), and participants were provided written informed consent before answering the survey questions. The last part of the informed consent read as, “By choosing "continue" on the question below, you are indicating that you voluntarily agree to participate in this survey.” And respondents who did not select “continue” would exit the survey.”

Line 101-105

“After providing consent and answering screening questions, respondents provided information relating to their snack and beverage consumption habits, attitudes towards health/environment, preferences for different healthy snack and beverage products, and behavioral and sociodemographic characteristics”

Lines 169-172

**Table 2. Definition of personality trait statements**

Personality trait

Innovativeness

- Compared to my friends, I purchase more new, different, or innovative food
- In general, I am amongst the first of my circle of friends to buy new, different, or innovative food.
- I buy new, different or innovative food before anyone else I know.
- Generally, I am amongst the first in my circle of friends to remember a brand of new, different or innovative food.
- If new, different, or innovative foods are available in shops and supermarkets I always purchase them.
- I do purchase new, different, or innovative foods even if I have not tasted/experienced them beforehand

Extraversion

- I enjoy human interaction
- I am enthusiastic
- I am talkative
- I am full of energy and I thrive on the presence of other people
- I take pleasure in activities that involve large social gathering
- I work well in a group
- I find few rewards in time spent alone
- I am bored when I am by myself

Note: Respondents’ agreement/disagreement with these statements was measured using the five-point Likert scale: 1= Strongly disagree, 2=Disagree, 3= Somewhat disagree, 4= Neither agree nor disagree, 5= Somewhat agree, 6=Agree, 7= Strongly agree

**Comment 7:** Also, kindly strengthen the discussion with additional relevant literature.

**Response to comment 7:** Thank you for your comment. We have added more text discussing the results in the context of previous studies, and also included additional citations throughout the discussion. For example –

Lines 341-346

“In line with our results, earlier studies of functional food found that purchase intention is higher when the health claims of the product are physiological (e.g., less risk of cardiovascular disease) rather than psychological (52–54). For instance, information about reduced cholesterol benefits resulted in an increase in consumers purchase intention for a fortified yoghurt drink (55), and in another study, information about antioxidants benefits increased consumers purchase intention for functional foods (56).”

Lines 352-356

“Some studies have examined the significance of psychological factors such as perceptions, beliefs, attitudes, trust and food neophobia (57–65). Our results show that Innovativeness is one factor that exhibits a strong relationship with the WTP for multiple products across the three product categories, which is reasonable considering the novelty of the products used in the study.”

Lines 384-387

“For the rest of the products, however, the respondents are not deterred by price. This conforms with Huang et al. (75) and Ares et al. (73) who argue that functional food consumers are usually more interested in the health benefits of the products than the price.”

**Response to Reviewer 1**

**Comment 1:** The authors have presented a graphical abstract, I don't think the journal requires a structured abstract, please confirm.

**Response to comment 1:** Thank you for your comment. We rewrote the abstract to meet PLOS ONE’s requirements.

**Comment 2:** The structure of introduction is really confusing, authors have presented a mix information in this section. i.e., in first paragraph authors are giving a background and in second paragraph they are giving the objectives of the study while in 3rd paragraph again the authors have presented details about the topic. The objectives of the study and conceptualization should be shared at end of introduction.

**Response to comment 2:** Thank you for your comment, and apologies for any confusion in the introduction. Following your suggestion, we have restructured the introduction and moved the objectives paragraph next to the contributions at the end of the section.

**Comment 3:** The quality of figures provided is really poor and its being very difficult to interpret the results.

**Response to comment 3:** Thank you for your comment. We have made new copies of the figures using the PLOS ONE tool, which significantly improved the quality of the figures.

**Comment 4:** Authors should provide high quality illustration

**Response to comment 4:** Thank you for your comment. As mentioned in the response to your third comment, we have made new copies of the figures using the PLOS ONE tool and this significantly improved the quality of the illustrations in the manuscript.

**Comment 5:** Authors should add a graphical abstract

**Response to comment 5:** Thank you but we are confused by this comment. In your comment 1, you said that “The authors have presented a graphical abstract….” but in this comment, you ask us to add a graphical abstract. We are really confused.

But anyways, based on your comment 1, we have provided an abstract that meets PLOS ONE requirements.

**Comment 6:** The conclusion part is missing in the main manuscript file

**Response to comment 6:** Thank you for your comment. The “Implications for Research and Practice” section was meant to serve as the conclusion. We have renamed this section to avoid confusion, and we also edited this section with more information about limitations and suggestions for future work.

Line 448

**“Conclusion/Implications for Research and Practice”**

Line 460-472

“Finally, despite the useful insights afforded in this study, it also has some limitations. First, consumer preferences were elicited using a stated preference approach, where the respondents reported their valuation in a hypothetical setting. This was necessary in this study since the products being investigated do not yet exist in the market. While we included a cheap talk script to improve the accuracy of the stated preferences, there is still a possibility of hypothetical bias occurring (where respondents report higher valuation than they would be willing to pay in a real shopping scenario). Future studies could investigate this point further by comparing preferences for similar products in a hypothetical vs incentive compatible setting. Second, our sample size is relatively small for a nationwide survey. Although the current sample size ensures meaningful statistical power for all analyses conducted in this study, it may not support in-depth sub-analyses over different consumer groups that vary in socioeconomic or behavioral characteristics. Future research could investigate these sub-analyses to potentially uncover important insights that could help identify segments of the market.”

**Response to Reviewer 2**

**Comment 1:** Discussion section may be improved by displaying more data already existing in the literature and compare them with those obtained in this study.

**Response to comment 1:** Thank you for your comment. We have added more text discussing the results in the context of previous studies, and also included additional citations throughout the discussion. For example –

Lines 341-346

“In line with our results, earlier studies of functional food found that purchase intention is higher when the health claims of the product are physiological (e.g., less risk of cardiovascular disease) rather than psychological (52–54). For instance, information about reduced cholesterol benefits resulted in an increase in consumers purchase intention for a fortified yoghurt drink (55), and in another study, information about antioxidants benefits increased consumers purchase intention for functional foods (56).”

Lines 352-356

“Some studies have examined the significance of psychological factors such as perceptions, beliefs, attitudes, trust and food neophobia (57–65). Our results show that Innovativeness is one factor that exhibits a strong relationship with the WTP for multiple products across the three product categories, which is reasonable considering the novelty of the products used in the study.”

Lines 384-387

“For the rest of the products, however, the respondents are not deterred by price. This conforms with Huang et al. (75) and Ares et al. (73) who argue that functional food consumers are usually more interested in the health benefits of the products than the price.”

**Comment 2:** In the conclusions, I recommend including the limitations observed while undertaking this investigation.

**Response to comment 2:** Thank you for your comments. We have included a paragraph on limitations

Line 460-472

“Finally, despite the useful insights afforded in this study, it also has some limitations. First, consumer preferences were elicited using a stated preference approach, where the respondents reported their valuation in a hypothetical setting. This was necessary in this study since the products being investigated do not yet exist in the market. While we included a cheap talk script to improve the accuracy of the stated preferences, there is still a possibility of hypothetical bias occurring (where respondents report higher valuation than they would be willing to pay in a real shopping scenario). Future studies could investigate this point further by comparing preferences for similar products in a hypothetical vs incentive compatible setting. Second, our sample size is relatively small for a nationwide survey. Although the current sample size ensures meaningful statistical power for all analyses conducted in this study, it may not support in-depth sub-analyses over different consumer groups that vary in socioeconomic or behavioral characteristics. Future research could investigate these sub-analyses to potentially uncover important insights that could help identify segments of the market.”
