# Supplementary material for: Perception and demand for healthy snacks/beverages among US consumers vary by product, health benefit, and color
Source: PLoS One. 2023 Jun 16;18(6):e0287232. doi: 10.1371/journal.pone.0287232 (PMC10275438; doi:10.1371/journal.pone.0287232)
Supplement: Attachment — Submitted filename: Response to Academic Editor and Reviewers_Final.docx [file pone.0287232.s004.docx]

**Response to Academic Editor and Reviewers**

**Response to Academic Editor**

**Comment 1:** Please, kindly address the comments raised by the reviewer, it will help improve the quality of this work.

**Response to comment 1:** Thank you for your comment. We have addressed the reviewer’s comments and included details below on revisions made to address the comments

**Response to Reviewer 3**

**Comment:** In general, the reviewer’s comments have been addressed well in the revised version. However, the introduction part must be improved: The flow of the introduction is not clear, why the study is needed and why it is important. Most of the introduction is about improving healthy snacks, not about consumer perception. While the study is investigating the consumers feedback. I would suggest focusing on consumer perceptions. For example, what is the current trend in the perception of potential consumers about developing new products (in general)? Then continue with the importance of healthy snacks, and continue to emphasize the current aim.

**Response to comment:** Thank you for your comment. We have incorporated your suggestions to improve the introduction section by adding discussions about consumer perceptions of new products (in general) and novel snacking products. We also reorganized the introduction to make the flow better.

Lines 71 – 109

There is a large body of work on consumer preferences for novel food technologies/products in general. In contrast to non-food domains, many technological innovations resulting in novel food products aren’t perceived as favorably by consumers (39–41). Given the importance of technological innovations to meeting global food demand, much research has been devoted to investigating the underlying factors driving these perceptions. Due to factors such as limited knowledge, consumers have been shown to utilize heuristics (e.g., emotions, trust, natural-is-better) in their evaluation of food products, which can result in biased decisions (39,42). The framing of food technology information can also influence consumers’ acceptance of new products (43–45). Additionally, food technology neophobia (46), disgust sensitivity (47), cultural differences (48), and personality traits such as openness and conscientiousness (49), may help explain some of the differences in consumer acceptance. Some other determinants revealed by various studies include food safety concerns (50), risk perceptions (50), socio-demographic factors (e.g., age, gender, education level) (51,52), lifestyle habits (e.g., vegetarian, travelling habit) (51,52). Our study contributes to this literature by exploring preferences specifically for novel healthy snacking products.

As demand for healthy snacking choices continues to increase, more research is focusing on developing new products that can appeal to consumers. Recent work has examined consumer perceptions of novel functional products such as fortified farmed fish (13), granola bars (14), enriched coffee (15), and probiotic yogurt (16), among others. Also, given the high nutritional value of vegetables and the growing global demand for plant-based food products (17), there is growing research into vegetable-based functional food product alternatives and general healthy snacking (18–20). Research shows that when consumers become more aware of their health benefits, they report a significant increase in the demand for these foods (21,22). For example, one study (23) found that Turkish consumers had a favorable attitude towards functional food products, with a majority believing that these foods are necessary and part of a healthy diet. Other studies across various countries found similar results using outcomes such as willingness to try (24,25), willingness to pay (26,27), and willingness to buy/purchase intention (28–31). These studies spanned various food and beverage products such as apples, tomatoes, yogurts, cereals, etc.

This stream of literature has identified a wide range of factors that possibly influence preferences for these novel products. Demographic factors such as age (13), gender (25), education level (32), and household size (32), and marital status (8) have been linked to willingness to consume various functional food products. The perceived healthiness of the product/ingredients (33), health information (27), knowledge of product brand (34), price (35), taste (31), and other product characteristics have also been found to influence preferences. Multiple studies have also reported associations between various psychological/behavioral characteristics such as, health consciousness (28), knowledge (36), trust (30), food neophobia (30), motivations (37), health-related behaviors (38), beliefs (38), and consumer preferences for novel food and beverage products
